# The Diagnosis and Treatment of Lumbago

**Published:** 1897-04

**Authors:** 


### THE DIAGNOSIS AND TREATMENT OF LUMBAGO.

F. H. Edgeworth, of Bristol, subdivides this disorder into three classes of cases,—those of rheumatic myalgia, rheumatic neuritis, and renal pain associated with hyperacidity of the urine,—and argues that, if the differential diagnosis be made, it is found that treatment is thereby much simplified and restoration to health more readily brought about.

The patients in the third class complain of a dull-aching pain in the lumbar region on both sides. The pain, which is constant and not made worse by movement, is not confined to the lumbar region; it extends forward around the trunk into the groins, and occasionally into the testes. The patient generally complains of scalding during micturition, and the urine, which is quite clear on first being passed, is very acid in reaction, and on cooling deposits abundant brick-red urates. The muscles are not tender on palpation or percussion, but there is often a zone of hyperæsthesia of the skin, best tested by prodding with the head of a pin, extending around from the lumbar region to the lower part of the trunk for a space of about three inches above Poupart's ligament and the pubes. The pain is evidently associated with and probably dependent on hyperacidity of the urine; just as scalding occurs during micturition from irritation of the urethra, so the bilateral lumbar and abdominal pains result from irritation of the renal pelves; and the hyperæthesia of the skin is a "referred" phenomenon exactly like that which happens in cases of renal calculus or gravel. The reflex retraction of the testis sometimes seen in renal calculus is not present in these cases, probably because the irritation is not sufficiently great; and the phenomena are always bilateral, though they may be more marked on one side than the other. Treatment is simple and most successful, consisting in the administration of large doses of some indirect antacid, such as potassium citrate.

Lumbago due to myalgia is characterized by pain in the lumbar region only, generally on both sides, though occasionally on one side only. It very rarely extends to the muscles of the anterior abdominal wall. When the patient is at rest or lying down, there is no pain, which occurs only during movement. An easy way of satisfying one's self as to this is to ask the patient to stoop down and touch his toes; the action is performed carefully and stiffly, with evident discomfort, often much greater while resuming the upright position than in stooping. The muscles are not, in the least, tender on palpation, and there is no hyperæthesia of the skin either over the affected muscles or on the front wall of the abdomen. The urine is normal, and there is no scalding during micturition.

Lumbago from rheumatic neuritis resembles rheumatic myalgia in many of its features, but differs in three particulars. The pain, although rendered worse by movement, persists in a less degree during rest. Further, there is some tenderness on palpation, particularly over the spots where the nerves run through fasciæ. And, lastly, pain is often felt along the course of the sciatic nerve or nerves as well as in the lumbar region. If a case be seen early there is rarely any evidence of impairment in the conductivity of the nerves.

The treatment of lumbago due to rheumatic myalgia or neuritis consists in the administration of salicylates until toxic effects are produced, and in the use of counter-irritation, the best application for which is capsicum, which was recommended many years ago, but has been but little used. The ordinary B. P. tincture is diluted with water in the proportion of 1 to 5, and applied on lint, covered by oiled silk or other protective to prevent evaporation. The effects of this are extraordinary and quite inexplicable,—a patient, who a quarter of an hour previously could not make the least movement by reason of a pain, is able to move freely and painlessly. This result is, it is true, somewhat transitory; it wears off in an hour or so, but can be re-induced by a fresh application. One usually directs this to be done for half an hour four or five times a day.

I have found that the salicylates act more speedily in cases of rheumatic neuritis than of rheumatic myalgia; in the former the pain usually ceases as soon as salicylism is produced, while this has to be kept up for some little time in cases of myalgia before its cessation.

These cases of rheumatic neuritis and myalgia have some etiological relationship with ordinary rheumatic arthritis. It is easy to divide cases of "rheumatism" into two great groups, in the first of which are such affections as endocarditis, pericarditis, nodules, chorea, and arthritis; and in the second, rheumatic myalgia and neuritis. Many authors assign them to different causes. And certainly such a division seems somewhat justifiable from a clinical point of view; as a rule, a patient suffers from one or more of the diseases of one group only. But such a division is a little arbitrary; if, for instance, a large number of cases of lumbago be taken, and those in which the pain is renal in origin be first subtracted, a large percentage of the remainder will be found to have suffered at one time or another from "major" rheumatic attacks.

The relationship, then, of these "minor" rheumatic affections is, perhaps, a good deal nearer that of ordinary rheumatism than is generally supposed, and affords an explanation of the good effects produced by the administration of salicylates.—*British Medico-Chirurgical Journal*, December, 1896.

